# Rabies virus phosphoprotein P5 binding to BECN1 regulates self-replication by BECN1-mediated autophagy signaling pathway

**DOI:** 10.1186/s12964-020-00644-4

**Published:** 2020-09-18

**Authors:** Juan Liu, Min Liao, Yan Yan, Hui Yang, Hailong Wang, Jiyong Zhou

**Affiliations:** 1 MOA Key Laboratory of Animal Virology, Center for Veterinary Sciences, Zhejiang University, 866 Yuhangtang Road, Hangzhou Zhejiang, 310058 PR China; 2 Collaborative innovation center and State Key laboratory for Diagnosis and Treatment of Infectious Diseases, The First Affiliated Hospital, Zhejiang University, Hangzhou, 310058 PR China

**Keywords:** Rabies virus phosphoprotein P5, Beclin1, Binding domain, Incomplete autophagy, Viral replication

## Abstract

**Background:**

Rabies virus (RABV) is reported to encode five phosphoproteins (P), which are involved in viral genomic replication, axonal transport, oxidative stress, interferon antagonism, and autophagy induction. However, the functions of the different P proteins are poorly understood.

**Methods:**

Immunofluorescence staining and western blot were performed to detect the autophagy activity, the form of ring-like structure, and the colocalization of BECN1 and P. Co-immunoprecipitation was performed to detect the interaction between P and BECN1. QRT-PCR and TCID_50_ assay were performed to detect the replication level of RABV. Small interfering RNA was used to detect the autophagy signaling pathway.

**Results:**

We found that P5 attaches to N-terminal residues 1–139 of BECN1 (beclin1) on the BECN1 ring-like structure through amino acid residues 173–222 of P5. Subsequently, we found that P5-induced autophagosomes did not fuse with lysosomes. *Becn1* silencing did not recover P5 overexpression-induced promotion of RABV replication. Mechanistically, RABV protein PΔN82 (P5) induced incomplete autophagy via the BECN1-mediated signaling pathway.

**Conclusions:**

Our data indicate that P5 binding to the BECN1 ring benefits RABV replication by inducing BECN1 signaling pathway-dependent incomplete autophagy, which provides a potential target for antiviral drugs against RABV.

Video abstract

**Graphical abstract:**

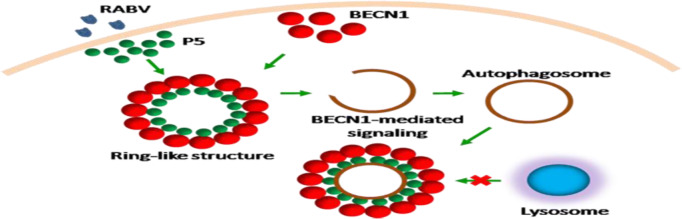

## Background

Rabies is associated with severe neurological symptoms and a high mortality rate, causing over 59,000 human deaths worldwide each year [1]. Rabies virus (RABV), belonging to the *Rhabdoviridae* family, is a single nonsegmented negative-stranded RNA virus with genome of 12 kb. The RABV genome encodes a nucleoprotein (N), phosphoprotein (P), matrix protein (M), glycoprotein (G), and RNA polymerase (L) [2]. The RABV M protein induces apoptosis by targeting mitochondria [3]. The viral protein P is a multifunctional protein that is involved in viral transcription and replication [4]. The P protein–dynein LC8 interaction is involved in the axonal transport of rabies virus along microtubules through neuronal cells [5]. The interaction of RABV P protein with complex I in mitochondria causes mitochondrial dysfunction, increased generation of reactive oxygen species (ROS), and oxidative stress [6]. In addition, the interaction of RABV P protein with the focal adhesion kinase and nucleolin positively regulates viral replication [7]. However, RABV P protein also directly binds to the interferon-induced promyelocytic leukemia protein, which inhibits viral replication [8]. P protein’s interaction with both cell division cycle 37 (CDC37) and heat shock protein 90 (HSP90) promoted self-stability [9]. RABV P binding to beclin1 (BECN1) can induce incomplete autophagy through the caspase2 (CASP2)-mediated signaling pathways to promote viral genome replication [10]. RABV P also interacts with signal transducer and activator of transcription 1 (STAT1) to counteract interferon (IFN) signaling by creating both cytoplasmic and nuclear blocks for STAT1 [11]. RABV P protein, via an interferon antagonist interaction with activated STAT3, inhibits membrane glycoprotein 130 (GP130) receptor signaling to generate optimal cellular conditions for viral replication and spread [12].

The RABV P protein is phosphorylated by a RABV protein kinase and protein kinase C, forming different phosphorylated versions of the P protein [13]. The full length P and small P proteins P2, P3, P4, and P5, are translated from downstream in-frame AUG initiation codons by a leaky scanning mechanism [14]. These small P proteins have different subcellular localizations. The small P proteins P3, P4, and P5 are located in the nucleus because of the nuclear localization signal (NLS) located in the C-terminal part of the protein (amino acids 172–297), whereas the cytoplasmic distributions of P and P2 result from a CRM1 nuclear export signal (NES) in the N-terminal part of the protein [15]. Thus, the functions of these small P proteins have not been fully determined.

In the present study, we aimed to determine the function of the small P protein PΔN82 (P5). The results showed that P5 binding to the BECN1 ring-like structure induced incomplete autophagy via the BECN1 mediated signaling pathway, which promotes RABV replication. Our study highlights the role of viral protein P5 in modulating RABV replication.

## Materials and methods

### Cells and viruses

Mouse neuroblastoma N2a cells (CCL-131) and human embryonic kidney 293 T cells (CRL-3216) from ATCC were maintained in Dulbecco’s modified Eagle’s medium (DMEM; Gibco, Carlsbad, CA, USA) supplemented with 10% heat-activated fetal bovine serum (Gibco/Invitrogen, Carlsbad, CA, USA), 100 U of penicillin mg/ml, and 100 mg of streptomycin/ml. RABV strains HEP-Flury and CVS-11 were stored in our laboratory and were propagated in N2a cells. Briefly, N2a cells were infected with RABV at a multiplicity of infection (MOI) of 2. The infected cells were grown in fresh medium at 37 °C and 5% CO_2_ for the indicated times.

### Antibodies and reagents

Rabbit anti-LC3A/B (4108), anti-p-AKT (Ser473) (4060), anti-AKT (4691), and anti-p-MTOR (Ser2448; 5536), anti-MTOR (2983), anti-p-AMPK (4185), anti- AMPK (2532), anti-p-ERK (4370), anti- ERK (4695), anti-p-P38 (4511), and anti-P38 (9212) antibodies were purchased from Cell Signaling Technology (Beverly, MA, USA). Rabbit anti-P62 (3340–1) antibody was purchased from Epitomics (Burlingame, CA, USA). Rabbit anti-CASP2 (ab179520), anti-ULK1 (ab128859), and anti-ATG5 (ab108327) antibodies were purchased from Abcam (Cambridge, MA, USA). Mouse anti-ATG7 (sc-376,212) and anti-BECN1 (sc-48,341) antibodies were purchased from Santa Cruz Biotechnology (Santa Cruz, CA, USA). Mouse anti-Flag (clone M2) (F1804) mAb, wortmannin (W1628), and 3-MA (M9281) were purchased from Sigma-Aldrich (St. Louis, MO, USA). Rabbit anti-MYC (R1208–1) and anti-GAPDH/glyceraldehyde-3-phosphate dehydrogenase (EM1101) antibodies were purchased from Huaan Biological Technology (Hangzhou, China). Mouse monoclonal antibodies (mAbs) recognizing the N/P proteins of RABV were produced in our laboratory [16]. Fluorescein isothiocyanate (FITC)-labeled goat anti-mouse IgG (62–6511) and Alexa Fluor 546-conjugated anti-rabbit IgG (A10036) were obtained from Thermo Scientific (Waltham, MA, USA). Secondary antibodies comprising horseradish peroxidase-labeled anti-mouse (074–1806) or anti-rabbit (074–1506) IgG were purchased from Kirkegaard & Perry Laboratories (Millford, MA, USA). Adenovirus expressing mCherry-GFP-LC3B fusion protein (C3011), cell lysis buffer NP-40 (50 mM Tris pH 7.4, 150 mM NaCl, 1% NP-40) (P0013F) and phenylmethyl sulfonylfluoride protease inhibitor (ST505) were purchased from Beyotime (Shanghai, China). Earle’s balanced salt solution (EBSS) (14155–063) was purchased from Gibco. The jetPRIME cell transfection kit (PT-114-07) was purchased from Polyplus Transfection (Sébastien Brant, Illkirch, France).

### Plasmids construction and transfection

The specific primers used to make the constructs generated in this study are listed in Table S1. The truncated *P* genes were amplified from the cDNA of HEP-Flury (Accession: AB085828.1) and cloned into pCMV-N-Flag (Clontech, Mountain View, CA, USA, 635688) or pCMV-N-Myc (Clontech, 635,689). The truncated *Becn1* genes were amplified from the cDNA of *Mus musculus Becn1* (Accession: NM_019584.3) and cloned into pCMV-N-Myc. The other plasmids Flag-P (full-length) and Myc-BECN1 were stored in our laboratory. The siRNA targeting mouse *Becn1* gene and shRNA targeting mouse *Akt*, *Mtor*, *Ampk*, *Mapk* genes were also synthesized by Genepharma (Suzhou, China). The siRNA or shRNA and all the plasmids were transfected into HEK293T or N2a cells using the jetPRIME kit according to the manufacturer’s instructions.

### Confocal microscopy

HEK293T or N2a cells were grown to 70% confluence on a confocal plate (In Vitro Scientific, Sunnyvale, CA, USA) and then transfected with the indicated plasmids or infected with RABV (MOI = 2) for 24 h. The cells were washed with phosphate-buffered saline (PBS), fixed, and permeabilized with 4% paraformaldehyde in PBS at 4 °C for 20 min, and then incubated with the appropriate primary and secondary antibodies. The nuclei were stained with 4, 6-diamidino-2-phenylindole (DAPI). Fluorescence signals were scanned under a Zeiss LSM 780 laser confocal microscopy (Zeiss, Oberkochen, Baden-Württemberg, Germany).

### Western blotting

Cells were harvested and lysed with NP-40 buffer containing phenylmethyl sulfonyl fluoride at 4 °C for 2 h. Protein samples were separated by 12% sodium dodecyl sulfate polyacrylamide-gel electrophoresis and were transferred onto nitrocellulose membranes. 5% nonfat dry milk containing 0.1% Tween 20 was used to block the nonspecific binding sites for 1 h at room temperature, and then the membranes were incubated with primary antibody at 4 °C overnight, followed by the appropriate secondary antibody for 1 h at room temperature. The blots were developed using a SuperSignal West Femto Substrate Trial Kit (Thermo Scientific, 34,096) according to the manufacturer’s protocol. Images were captured using FluorChemm M chemiluminescent imaging system (Protein Simple, Santa Clara, CA, USA) and ImageJ software (National Institutes of Health, Bethesda, MD, USA) was used to quantify the intensity of the immunoreactive protein bands.

### Co-immunoprecipitation

Transfected HEK293T cells were lysed for 2 h with NP-40 buffer containing 1 mM phenylmethyl sulfonylfluoride protease inhibitor at 4 °C. The insoluble fractions were removed after the cell lysates were centrifuged at 12,000 *g* for 10 min at 4 °C. The soluble fractions were incubated with the indicated antibodies overnight at 4 °C. Fresh protein A/G agarose (Santa Cruz Biotechnology, sc-2003) was then added at 4 °C for 8 h before washing with PBS. The eluted proteins were subjected to western blotting analysis.

### QRT-PCR

Quantitative real-time reverse transcription polymerase chain reaction (qRT-PCR) analyses of the mRNA transcripts and anti-genomic RNA were performed as previously described [17]. Briefly, Total RNA was extracted from N2a cells using the TRIZOL Reagent (SuperfecTRI™, 3101–100; Shanghai Pufei Biotech Co., Ltd., Shanghai, China) according to the manufacturer’s instructions. The total RNA was reverse transcribed into cDNAs using a RevertAid RT Reverse Transcription Kit (Thermo Scientific, K1691). The qRT-PCR specific primers used are as follows: upstream primer 5′-AAGGAGTTGAATGACAGGGTGCCA-3′ and downstream primer 5′-ACT TGGGATGGTTCGAAAGGAGGA-3′ for the RABV anti-genome (115 bp in length), upstream primer 5′-AGCAGCAATGCAGTTCTTTGAGGG-3′ and downstream primer 5′-TTGTCAGTTCCATGCCTCCTGT CA-3′ for the RABV *N* gene (164 bp in length), and upstream primer 5′ -TCAACAGCAACTCCCACTCTTCCA-3′ and downstream primer5′-ACCCTGTTGCTGTAGCCGTATTCA-3′ for *Gapdh* gene (92 bp in length). The qPCR reaction was performed using ChamQ™ Universal SYBR® qPCR Master Mix (Vazyme Biotechnology, Q711–02, Nanjing, China) and a LightCycler96 real-time PCR system (Roche, Basel, Switzerland). The qPCR conditions were as follows: 95 °C for 300 s; 40 cycles of 95 °C for 10 s and 60 °C for 30 s; 95 °C for 10 s, 65 °C for 60 s and 97 °C for 1 s; 37 °C for 30 s. Quantitative analysis was performed using the LightCycler96 software with a relative quantification method (ΔΔCt) to analyze the levels of viral *N* mRNA and anti-genomic RNA.

### Statistical analysis

Statistically significant differences between groups were determined using one-way analysis of variance (ANOVA) with the Tukey Multiple Comparison Test and using GraphPad Prism 5 software (GraphPad Software, Inc., La Jolla, CA, USA). A *P* value of less than 0.05 was considered statistically significant.

## Results

### Residues 173–222 of the RABV P protein form an autophagy-inducing domain

**Fig. 1:**
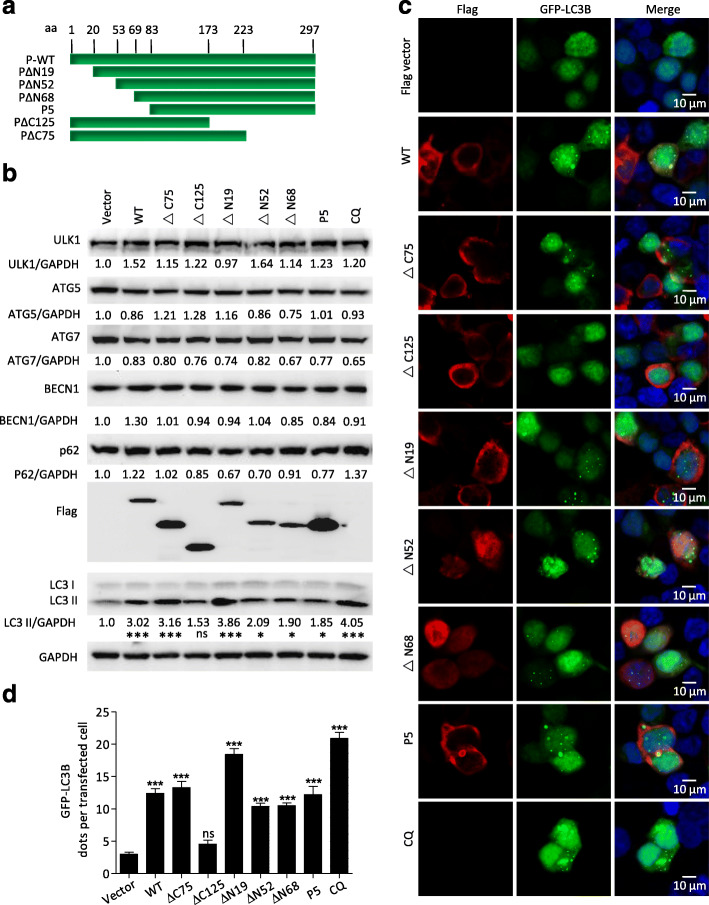
Truncated P proteins induce autophagy. **a** Schematic diagram of the full-length and truncated P proteins. **b** HEK293T Cells were transfected with the plasmids containing the truncated *P* genes for 48 h, harvested, and analyzed using western blotting using mouse anti-ATG7, anti-Flag mAb, rabbit anti-LC3A/B, anti-ATG5, anti-BECN1, anti-P62, anti-ULK1, and anti-GAPDH antibodies. **c** HEK293T cells were cotransfected with GFP-LC3B and the plasmids containing the truncated *P* genes for 24 h, fixed, and immunostained with mouse anti-Flag antibodies (red), and then visualized using confocal microscopy. DAPI (blue) was used to stain nuclear DNA. Scale bar: 10 μm. **d** The graph shows the quantification of autophagosomes by taking the average number of dots in 50 cells. Means and SD (error bars) of three independent experiments are indicated (*, *P* < 0.05; **, *P* < 0.01; ***, *P* < 0.001)

Our previous report discovers that the RABV P protein induces incomplete autophagy [10], however, the domain responsible for this incomplete autophagy induction is unknown. To identify the functional domain of the P protein required for autophagy induction, we constructed six truncated P protein mutants (Fig. 1a). 293 T cells were transfected with the plasmids pCMV-N-Flag-tagged P, PΔC75, PΔC125, PΔN19 (P2), PΔN52 (P3), PΔN68 (P4), and PΔN82 (P5), respectively. Western blotting assay revealed that the level of endogenous LC3-phosphatidylethanolamine conjugate (LC3-II) was dramatically increased in cells transfected with all truncated P mutants except for those transfected with PΔC125 compared with the empty vector transfected cells, and chloroquine (CQ), a lysosomal proteolysis inhibitor, as a control for autophagic flux (Fig. 1b; *P* < 0.05, *P* < 0.001). Moreover, all truncated P proteins caused no significant increases in autophagy associated proteins autophagy related (ATG)5, ATG7, Unc-51 like autophagy activating kinase 1 (ULK1), BECN1, and autophagic degradation substrate sequestome 1 (also known as P62) levels (Fig. 1b). Consistently, confocal observation also revealed green fluorescent protein (GFP)-LC3B punctas were dramatically increased in cells transfected with all truncated P mutants except for those transfected with PΔC125 compared with the empty vector transfected cells (Fig. 1c and d; *P* < 0.001), however, there were no significant differences in GFP-LC3B punctas caused by all truncated P proteins after these cells were treated with CQ (Fig. S1). Therefore, these data showed that a P protein containing C-terminal residues aa 173–222 was responsible for autophagic activity.

### Phosphoprotein P5 forming ring-like structures induces autophagosomes accumulation

**Fig. 2:**
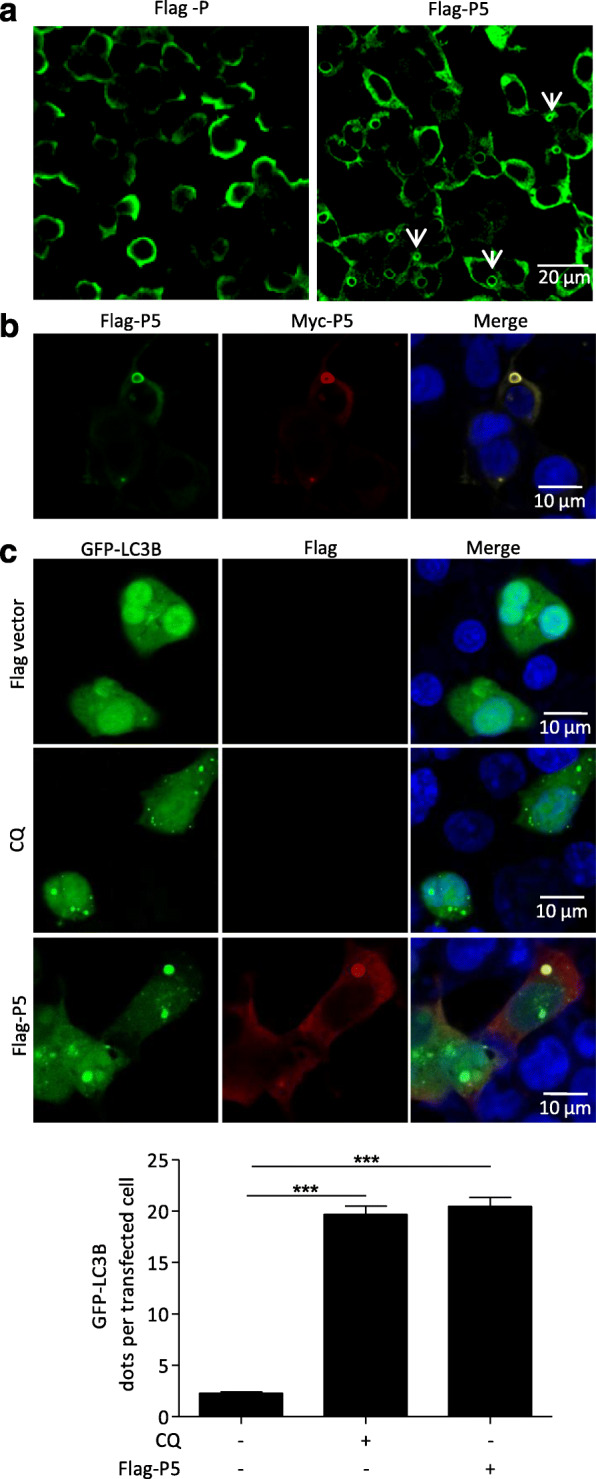
Accumulated autophagosomes are surrounded by a ring-like structure comprising P5. **a-c** N2a cells were transfected with Flag-P or Flag-P5, or cotransfected with Flag-P5 and Myc-P5 or GFP-LC3B plasmids for 24 h, fixed, and immunostained with mouse anti-Flag antibodies and rabbit anti-Myc antibodies, and then visualized using confocal microscopy. DAPI (blue) was used to stain nuclear DNA. Scale bar: 10 or 20 μm. The graph shows the quantification of autophagosomes by taking the average number of dots in 50 cells. Means and SD (error bars) of three independent experiments are indicated (*, *P* < 0.05; **, *P* < 0.01; ***, *P* < 0.001)

Small phosphoprotein P5 contains residues aa 83–172, 173–222, and 223–297 of full-length P protein. Interestingly, a ring circle-like structure was observed in N2a cells transfected only with the P5 mutant but not in N2a cells either transfected individually with other P protein mutants or co-transfected with P5 and other P protein mutants (Fig. 1c, Fig. 2a and Fig. S2). Similarly, the ring circle-like structure was observed in N2a cells co-transfected with both Flag-P5 and Myc-P5 (Fig. 2b), indicating that only P5 could form the ring-like structure. Moreover, the number of GFP-LC3B puncta autophagosomes surrounded by the P5 ring-like structure increased significantly in N2a cells cotransfected with GFP-LC3B and Flag-P5 in comparison with N2a cells cotransfected with Flag-vector and GFP-LC3B (Fig. 2c). Collectively, these data demonstrated that the P5 ring-like structure induced autophagosomes accumulation.

### Incomplete autophagic vesicles are induced by P5 protein

**Fig. 3:**
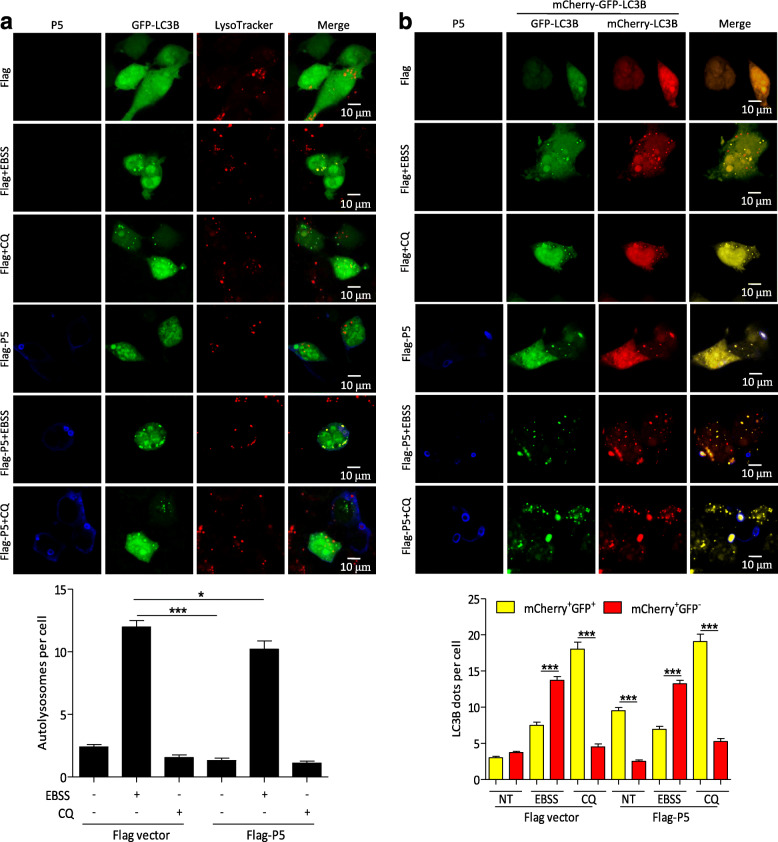
Autophagosomes fail to fuse with lysosomes in Flag-P5-transfected cells. **a** N2a cells were cotransfected with Flag-P5 and GFP-LC3B for 24 h, and were treated with EBSS or CQ for 4 h. Cells were incubated with LysoTracker Red (50 nM) for 15 min, and then were fixed, and immunostained with mouse anti-Flag mAb (blue), and observed using confocal microscopy to analyze fusion of autophagosomes with lysosomes. Scale bars: 10 μm. The graph shows the quantification of autolysosomes by taking the average number of dots in 50 cells. **b** N2a cells were transfected with Flag-P5 for 8 h, and infected with the adenovirus expressing mCherry-GFP-LC3B protein for 24 h, and were treated with EBSS or CQ for 4 h. Cells were fixed, and immunostained with mouse anti-Flag mAb (blue), and observed using confocal microscopy to analyze fusion of autophagosomes with lysosomes. Scale bars: 10 μm. The graph shows the quantification of autophagosomes by taking the average numberof dots in 50 cells. Means and SD (error bars) of three independent experiments are indicated (*, *P* < 0.05; **, *P* < 0.01; ***, *P* < 0.001)

To further investigate the relationship between autophagosomes and P5, N2a cells were cotransfected with Flag-P5 and GFP-LC3B, and labeled with LysoTracker Red. As expected, the number of GFP-LC3B puncta autophagosomes increased markedly and did not colocalize with LysoTracker Red in Flag-P5-transfected N2a cells compared with EBSS-treated N2a cells (Fig. 3a), indicating that the autophagosomes did not fuse with acidic compartments after P5 transfection. To rule out the possibility that autophagosomes fused with lysosomes but were not efficiently acidified in the transfected cells, we investigated the colocalization of GFP-LC3B with lysosomal associated membrane protein 1 (LAMP1) in Flag-P5-transfected N2a cells. GFP-LC3B puncta did not colocalize with LAMP1 in Flag-P5-transfected N2a (Fig. S3). These data suggested that autophagosomes did not efficiently fuse with lysosomes in Flag-P5-transfected cells.

In addition, we used adenovirus that expressed mCherry-GFP-LC3B, which was used to discriminate autophagosomes (expressing both mCherry and GFP fluorescent) from acidified autolysosomes (expressing red fluorescentonly) to determine the function of P5 in autophagosome maturation. N2a cells were transfected with Flag-P5 plasmids for 12 h, and infected with the adenovirus. In Flag vector transfected cells, few yellow puncta autophagosomes could be detected after adenovirus infection (Fig. 3b). In contrast, in Flag-P5 transfected cells, we observed the accumulation of yellow puncta autophagosomes but a low number of mcherry puncta autophagosomes, suggesting impaired autophagosome fusion with lysosomes. These results implied that P5 protein was responsible for the observed incomplete autophagic induction.

### The protein P5 attaches to the BECN1 ring-like structure by interaction with BECN1

**Fig. 4:**
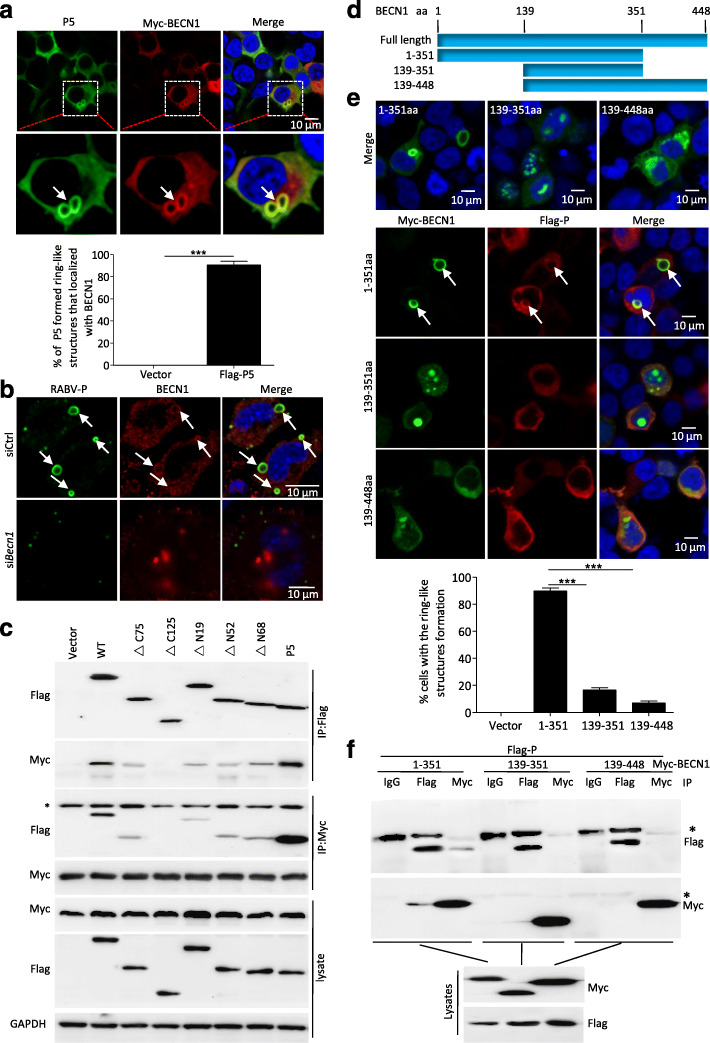
Truncated protein P5 interacted with BECN1. **a**, **b** N2a cells were cotransfected with Flag-P5 and Myc-BECN1 (**a**) or singly transfected with si*Becn1* RNA for 12 h and infected with RABV strain HEP-Flury at MOI = 2 for 24 h (**b**), and the viral P/P5 protein (green), BECN1 (red), and DAPI (blue) were detected using the indicated antibodies via confocal microscopy. White arrows indicate the colocalization of the ring-like structure. Scale bar: 10 μm. The graph shows the quantification of the percentage of BECN1 localization with P5 ring-like structure. **c** HEK 293 T cells were cotransfected with the plasmids containing the truncated *P* genes and Myc-BECN1 for 48 h, and the interactions between the truncated P protein and BECN1 were determined using the indicated antibodies. IP, immunoprecipitation. The asterisk indicates the heavy chains. **d** Schematic diagram of the full-length and truncated BECN1 proteins. **e** N2a cells were singly transfected or cotransfected with the plasmids encoding the truncated BECN1 genes and Flag-P for 24 h, and the viral P protein (red), BECN1 (green), and DAPI (blue) were detected using the indicated antibodies via confocal microscopy. White arrows indicate the colocalization of the ring-like structure. Scale bar: 10 μm. The graph shows the quantification of the ring-like structures formed with BECN1 deletion mutants. **f** HEK 293 T cells were cotransfected with the plasmids containing the truncated *Becn1* genes and Flag-P for 48 h, and the interactions between the truncated BECN1 proteins and P were determined using the indicated antibodies. IP, immunoprecipitation. The asterisk indicates the heavy chains. Means and SD (error bars) of three independent experiments are indicated (***, *P* < 0.001)

We previously demonstrated that the RABV P protein could interact with BECN1 [10]. To identify whether BECN1 binding to the P protein involves P5, N2a cells were cotransfected with Myc-BECN1 and Flag-P5 or Flag-PΔC75, Flag-PΔC125, Flag-PΔN19, Flag-PΔN52, and Flag-PΔN68, respectively. Confocal microscopy showed that the BECN1 colocalized with the full-length P and the P mutants except for PΔC125P5 mutant, notably, the P5 formed ring-like structure had stronger localization with BECN1 ring-like structure compared with P ring-like structure (Fig. 4a, and Fig. S4). Similarly, endogenous P protein colocalized with BECN1 to form the ring-like structure in RABV infected cells, and interestingly, the ring-like structure was not observed after *Becn1* gene was knocked down, suggesting that BECN1 was necessary for RABV infection to form the ring-like structure (Fig. 4b). Subsequently, a co-immunoprecipitation assay (Co-IP) was performed to further analyze whether the colocalization involves protein-protein interactions. The Co-IP data demonstrated that full-length and all truncated P proteins except PΔC125 could immunoprecipitate BECN1, and that P5 showed stronger binding to BECN1 than the other truncated P mutants (Fig. 4c). In addition, surprisingly, P5’s binding ability to BECN1 was stronger than that of the full-length protein (Fig. 4c). To identify the P protein binding domain of BECN1, Myc-tagged truncation mutants of BECN1 (1–351aa, 139–351aa, and 139–448aa) were constructed and transfected into 293 T cells (Fig. 4d). Confocal microscopy analysis showed that only the 1–351aa BECN1 mutant formed the ring-like structure, and the P protein colocalized with the ring-like structure and the 1–351aa BECN1 mutant (Fig. 4e). Further co-IP experiments showed that only 1–351aa BECN1, but not 139–351aa BECN1 and 139–448aa BECN1, interacted with P protein (Fig. 4f), revealing that first 139 N-terminal residues of BECN1 are responsible for interacting with P. Collectively, these data confirmed that RABV protein P attached to the BECN1 ring-like structure by residues 173–222 of P binding to N-terminal residues 1–139 of BECN1.

### P5 binding to BECN1 ring-like structure promoted RABV replication

**Fig. 5:**
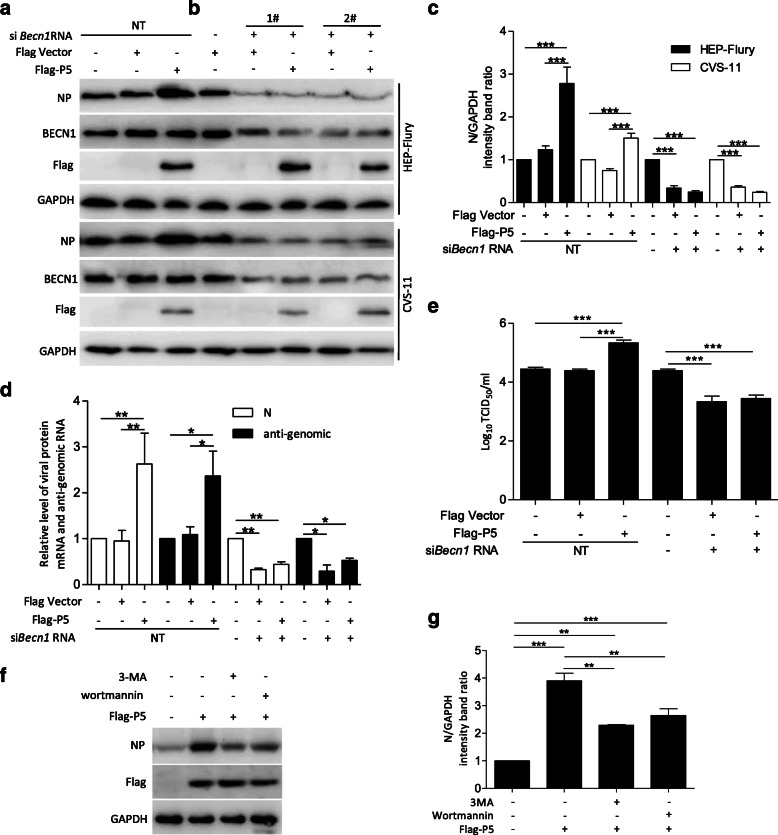
A ring-like structure containing P5 was beneficial to virus replication. **a**, **b** N2a cells were transfected with Flag-P5 (**a**) or cotransfected with Flag-P5 and two siRNAs targeting *Becn1* (**b**) for 12 h, and infected with RABV strain HEP-Flury or CVS-11 at an MOI = 2 for 36 h, and then harvested for western blotting analysis with mouse anti-NP, anti-BECN1, anti-Flag mAbs, and rabbit anti-GAPDH antibodies. **c** The ratio of viral protein N to GAPDH was normalized to that of the control conditions. **d** N2a cells were treated as described for panels A and B. qRT-PCR analysis of cellular viral *N* mRNA, and the anti-genomic RNA level. All qRT-PCR data were normalized to the expression of *Gapdh* and the control group was used as a reference. **e** Cellular supernatant in panels (**a** and **b**) were harvested for virus titer detection. Virus titers in N2a cells were determined using a TCID_50_ assay. **f** N2a cells were transfected with Flag-P5 for 12 h and were pretreated with 5 mM 3-MA or 1 mM wortmannin for 2 h, and then infected with RABV HEP-Flury strain at an MOI = 2 and incubated in the absence or presence of 5 mM 3-MA or 1 mM wortmannin for 24 h, and the cells were harvested for western blotting analysis using the indicated antibodies. **g** The ratio of viral protein N to GAPDH was normalized to that of the control conditions. Means and SD (error bars) of three independent experiments are indicated (*, *P* < 0.05; **, *P* < 0.01; ***, *P* < 0.001)

To determine the effect on RABV replication of P5 binding to the BECN1 ring-like structure, we investigated the dynamics of RABV infection under condition of P5 overexpression. N2a cells transfected with Flag-P5 for 12 h were infected with RABV. We found that the level of viral N protein, viral *N* mRNA, viral anti-genomic RNA, and infectious RABV progeny were all significantly increased; however, when *Becn1* was knocked down using two short interfering RNAs (si*Becn1*), there was a detectable downregulation of viral N protein, viral *N* mRNA, viral anti-genomic RNA, and infectious RABV progeny in the absence or presence of P5 (Fig. 5a-e, *P* < 0.05, 0.01, or 0.001), suggesting a positive role of P5 in regulating RABV infection dependent of BECN1. In addition, to further confirm whether the effect of the ring-like structure on RABV replication was dependent of autophagy induction, we also detected the level of viral N protein in presence of protein P5 together with the autophagy inhibitor 3-methyladenine (3-MA), or wortmannin treatment. The results showed 3-MA or wortmannin treatment significantly inhibited the level of viral N protein compared with that in non-treated P5 group (Fig. 5f and g, *P* < 0.01 or 0.001). Collectively, these data demonstrated that RABV replication hijacked BECN1 by P5 binding to the BECN1 ring-like structure.

### P5 binding to the BECN1 ring-like structure regulates RABV replication via the BECN1-mediated signaling pathway

**Fig. 6:**
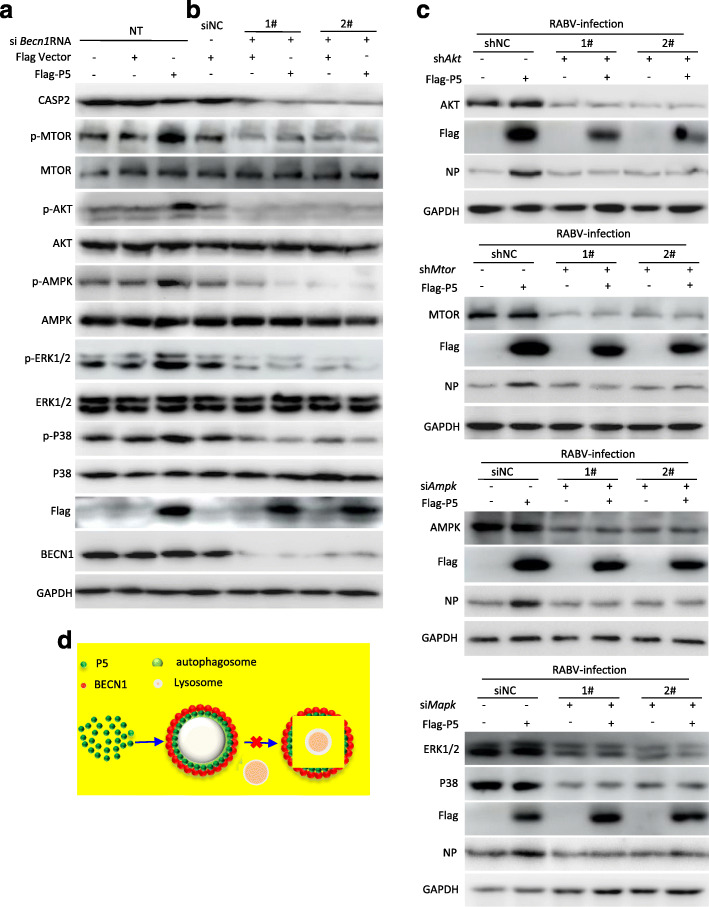
The protein P5 binding to the BECN1 ring-like structure regulates RABV replication via the BECN1-mediated signaling pathway. **a**, **b** N2a cells were transfected with Flag-P5 (**a**) or cotransfected with Flag-P5 and two siRNAs targeting *Becn1* (**b**) for 48 h, and then harvested for western blotting analysis with the indicated antibodies. **c** N2a cells were cotransfected with Flag-P5 and the shRNA/siRNA targeting *Akt* or *Mtor* or *Ampk* or *Mapk* for 12 h, and infected with RABV HEP-Flury strain at an MOI = 2 for 36 h. Cells were harvested for western blotting analysis with the indicated antibodies. **d** Proposed model showing how P5 protein regulates RABV replication. Data are representative of 3 independent experiments

To further investigate the BECN1-dependent signaling pathway through which P5 regulates RABV replication, we examined whether BECN1, AMP-activated protein kinase (AMPK), CASP2, protein kinase B (AKT), mammalian target of rapamycin (MTOR), and mitogen activated protein kinases MAPKs [extracellular signal-regulated kinase (ERK), P38] levels changed during overexpression of P5. Western blotting analysis showed that P5 dramatically upregulated the phosphorylation (p) level of AKT, MTOR, AMPK, ERK1/2, and P38, and reduced the CASP2 level; however, it did not affect the total amount of these proteins nor BECN1 levels (Fig. 6a). Moreover, we knocked down cellular *Becn1* using si*Becn1* to further show whether the P5 protein affected the expression of BECN1, AMPK, CASP2, AKT, MTOR, and MAPK (ERK, P38). The results showed that there was a significant downregulation of CASP2 and p-AMPK, p-AKT, p-MTOR, and p-MAPK (ERK1/2, P38) levels, and an insignificant alteration of the total amount of these proteins in *Becn1*-knockdown cells with viral gene *P5* transfection (Fig. 6b).

To investigate whether P5 regulated RABV replication depends on the downstream of BECN1-dependent signaling pathway, next we knocked down cellular *Akt*, *Mtor*, *Ampk*, *Mapk* respectively, and examined the NP expression levels in the absence or presence of P5. The results showed that there was a significant decrease of NP in absence or presence of P5, suggesting that the RABV replication was dependent of the AKT, MTOR, AMPK, MAPK proteins (Fig. 6c). Collectively, these data demonstrated that the BECN1 binding to P5 was responsible for regulating RABV replication via a BECN1-mediated signaling pathway.

## Discussion

Previous studies showed that full-length P protein and at least four additional shorter products P2 (PΔN19), P3 (PΔN52), P4 (PΔN68), and P5 were detected in RABV-infected cells, viral gene *P* transfected cells, and purified RABV virions [14]. BECN1 plays an interacting partner role for the mammalian phosphatidylinositol 3-kinase catalytic subunit type 3 (PIK3C3) involving macroautophagy, in which it is an essential chaperone or adaptor [18–20]. However, in the relationship between the virus and autophagy, although it has been reported that BECN1 interacts with a virus protein to regulate autophagy, the specific domain responsible for the BECN1 interaction is not clear [21, 22]. The present study showed that BECN1 exists in a ring-like structure, and identified that among five truncated P proteins (PΔC75, PΔN19, PΔN52, PΔN68, and P5), residues 173–222 induced autophagy by interacting with N-terminal residues 1–139 of BECN1. Meanwhile, only the full-length P protein and P5 were visibly colocalized with the BECN1 ring-like structure (Fig. 4). Notably, in co-IP experiments, P5 showed stronger binding than full length P protein. Therefore, we concluded that RABV small phosphoprotein P5 is responsible for binding to the BECN1 ring-like structure.

As an essential cofactor of RABV RNA polymerase, P may participate in additional physiological processes [23]. Our previous research reports incomplete autophagy induced by the RABV phosphoprotein [10]. In this study, we demonstrated that the P proteins with, but not without, amino acid segment 173–222 are involved in increasing the level of endogenous lipidated LC3-II. In particular, the LC3-II was surrounded by the P5 protein (Figs. 1 and 2). However, P5 did not change the levels of autophagy associated proteins ATG5, ATG7, ULK1, BECN1, and P62, markedly. In addition, the P5-induced autophagosome did not colocalize with lysosomes (Figs. 1, 2 and 3 and S2). Nonetheless, we observed that P5 upregulated the phosphorylation of AMPK, MAPK (P38, ERK1/2), AKT, and MTOR, and decreased BECN1-dependent CASP2 levels (Fig. 6). Collectively, our data demonstrated that amino acid residues 173–222 of the viral P protein are responsible for inducing incomplete autophagy, and the binding of P5 to the BECN1 ring-like structure induced this incomplete autophagy by activating the BECN1 signaling pathway.

Autophagy can remove intracellular pathogens, including bacteria and viruses, by activating various cellular defense responses, including direct digestion of intracytoplasmic virions [21, 24], recognition of pathogen-associated molecular patterns through the delivery of viral genomes to endosomal toll-like receptors [25], activation of innate immune signaling [26], and regulation of the inflammatory response [27–31]. However, many viruses also subvert the autophagic machinery to enhance viral replication [32–37]. In this study, we demonstrated that P5 overexpression increased the level of viral N protein, viral *N* mRNA, viral anti-genomic RNA, and infectious RABV progeny, and these indexes were significantly inhibited in the absence or presence of P5 and knockdown of *Becn1* (Fig. 5). In addition, we also demonstrated that the P5 still increased the RABV replication when autophagy was inhibited (Fig. 5). These results suggested that RABV replication was regulated by the binding of P5 to the BECN1 ring-like structure. In our previous study, RABV P binding to BECN1 can induce incomplete autophagy through the pathways BECN1-CASP2-AMPK-MAPK and BECN1-CASP2-AMPK-AKT-MTOR and RABV-induced incomplete autophagy provides the scaffolds for the replication of RABV genome. However, in this study, we found that small phosphoprotein P5 binding to BECN1 formed a ring-like structure to induce incomplete autophagy through a BECN1 signaling pathway. The ring-like structure wrapped the immature autophagic vesicles and might prevent the fusion of autophagic vesicles and lysosomes from degrading the RABV, and thus benefited self-replication.

## Conclusion

In conclusion, we identified the binding domain between the RABV phosphoprotein and beclin1, and found that RABV P5 protein interacted with the BECN1 ring-like structure to induce incomplete autophagy through a BECN1 signaling pathway. P5 attached to the BECN1 ring-like structure promoted RABV self-replication (Fig. 6d). Thus, the results of the present study identified potential antiviral drug targets against RABV.

## Supplementary information

**Additional file 1: Table S1.** Primer used for the truncated P or BECN1 protein constructs.

**Additional file 2: Figure S1.** HEK293T cells were cotransfected with GFP-LC3B and the plasmids containing the truncated *P* genes for 24 h, and further treated with CQ for 4 h. These cells were fixed, and immunostained with mouse anti-Flag antibodies (red), and then visualized using confocal microscopy. DAPI (blue) was used to stain nuclear DNA. Scale bar: 10 μm. The graph shows the quantification of autophagosomes by taking the average number of dots in 50 cells. Means and SD (error bars) of three independent experiments are indicated (*, *P* < 0.05. **Figure S2.** The truncated protein P5 is required for a ring-like structure. N2a cells were cotransfected with Flag and Myc tagged plasmids encoding the truncated *P* genes for 24 h, fixed, and immunostained with mouse anti-Flag antibody (green) and rabbit anti-Myc (red), and then visualized by confocal microscopy. DAPI (blue) stained nuclear DNA. Scale bar: 10 μm. **Figure S3.** Autophagosomes fail to fuse with lysosomes in Flag-P5-transfected cells. N2a cells were cotransfected with Flag-P5 and GFP-LC3B for 24 h, and were treated with EBSS or CQ for 4 h. Cells were fixed, and immunostained with rabbit anti-LAMP1 mAb (red), and mouse anti-Flag mAb (blue), and observed using confocal microscopy to analyze fusion of autophagosomes with lysosomes. Scale bar: 10 μm. The graph shows the quantification of autolysosomes by taking the average number of dots in 50 cells. Means and SD (error bars) of three independent experiments are indicated (*, *P* < 0.05; **, *P* < 0.01; ***, *P* < 0.001). **Figure S4.** The truncated P proteins colocalize with BECN1. N2a cells were cotransfected with the plasmids encoding the truncated *P* genes and Myc-BECN1 for 24 h, and Flag (green), BECN1 (red) and DAPI (blue) were detected by using the indicated antibodies in confocal microscopy. Scale bar: 10 μm.

## Data Availability

The datasets used and/or analysed during the current study are available from the corresponding author on reasonable request.

## Abbreviations

AKT: Protein kinase B
AMPK: AMP-activated protein kinase
ANOVA: Analysis of variance
ATG: Autophagy related
BECN1: Beclin1
CASP2: Caspase2
Co-IP: Co-immunoprecipitation
DAPI: 6-diamidino-2-phenylindole
ERK: Extracellular signal-regulated kinase
GAPDH: Glyceraldehyde-3-phosphate dehydrogenase
GFP: Green fluorescent protein
LAMP1: Lysosomal associated membrane protein 1
LC3: Microtubule associated protein 1 light chain 3 alpha
mAbs: Mouse monoclonal antibodies
MAPKs: Mitogen activated protein kinases
MOI: Multiplicity of infection
MTOR: Mammalian target of rapamycin
P: Phosphoprotein
RABV: Rabies virus
siRNA: Small interfering RNA
