# Supplementary material for: Rabies virus phosphoprotein P5 binding to BECN1 regulates self-replication by BECN1-mediated autophagy signaling pathway
Source: Cell Commun Signal. 2020 Sep 18;18:153. doi: 10.1186/s12964-020-00644-4 (PMC7499888; doi:10.1186/s12964-020-00644-4)
Supplement: Supplementary file 2 — Additional file 1: Table S1. Primer used for the truncated P or BECN1 protein constructs. [file 12964_2020_644_MOESM2_ESM.docx]

| Constructs | Plasmids | Upstream primer | Downstream primer |
| --- | --- | --- | --- |
| pCMV-N-Flag-P(∆C75)  pCMV-N-Flag-P(∆C125)  pCMV-N-Flag-P(∆N19)  pCMV-N-Flag-P(∆N52)  pCMV-N-Flag-P(∆N68)  pCMV-N-Flag-P5  pCMV-N-Myc-P(∆N19)  pCMV-N-Myc-P(∆N52)  pCMV-N-Myc-P(∆N68)  pCMV-N-Myc-P5  pCMV-N-Myc-BECN1(1-351aa)  pCMV-N-Myc-BECN1(139-351aa)  pCMV-N-Myc-BECN1(139-448aa) | pCMV-N-Flag  pCMV-N-Flag  pCMV-N-Flag  pCMV-N-Flag  pCMV-N-Flag  pCMV-N-Flag  pCMV-N-Myc  pCMV-N-Myc  pCMV-N-Myc  pCMV-N-Myc  pCMV-N-Myc  pCMV-N-Myc  pCMV-N-Myc | 5′-CGGAATTCCGATGAGCAAGATCTTTGTTAATCC-3′  5′-CGGAATTCCGATGAGCAAGATCTTTGTTAATCC-3′  5′-CGGAATTCCGATGGCCGAAGAGACTGTTGAT-3′  5′-CGGAATTCCGATGAGGCAATTTCACCTGGAC-3′  5′-CGGAATTCCGATGGTTAGGGTGGGCGAAG-3′  5′-CGGAATTCCGATGAATGAGGGAGAGGACCC-3′  5′-CGGAATTCCGATGGCCGAAGAGACTGTTGAT-3′  5′-CGGAATTCCGATGAGGCAATTTCACCTGGAC-3′  5′-CGGAATTCCGATGGTTAGGGTGGGCGAAG-3′  5′-CGGAATTCCGATGAATGAGGGAGAGGACCC-3′  5′-CGGAATTCCGATGGAGGGGTCTAAGGCGTCC-3′  5′-CGGAATTCCGATGACAGATACTCTCTTAGACCAAC-3′  5′-CGGAATTCCGATGACAGATACTCTCTTAGACCAAC-3′ | 5′-CCCTCGAGGTTATATTCCTGAAGATCGAGAAG-3′  5′-CCCTCGAGGTTATCCGGCTTTTGAAGGTT-3′  5′-CCCTCGAGGTTAGCATGATGTGTAGCGATC-3′  5′-CCCTCGAGGTTAGCATGATGTGTAGCGATC-3′  5′-CCCTCGAGGTTAGCATGATGTGTAGCGATC-3′  5′-CCCTCGAGGTTAGCATGATGTGTAGCGATC-3′  5′-CCCTCGAGGTTAGCATGATGTGTAGCGATC-3′  5′-CCCTCGAGGTTAGCATGATGTGTAGCGATC-3′  5′-CCCTCGAGGTTAGCATGATGTGTAGCGATC-3′  5′-CCCTCGAGGTTAGCATGATGTGTAGCGATC-3′  5′-CCGCTCGAGTCAACAGTATAATGGCAACTCC-3′  5′-CCGCTCGAGTCAACAGTATAATGGCAACTCC-3′  5′-CCGCTCGAGTCACTTGTTATAGAACTGTGAG-3′ |

Table S1 Primer used for the truncated P or BECN1 protein constructs
