# Supplementary material for: Rabies virus phosphoprotein P5 binding to BECN1 regulates self-replication by BECN1-mediated autophagy signaling pathway
Source: Cell Commun Signal. 2020 Sep 18;18:153. doi: 10.1186/s12964-020-00644-4 (PMC7499888; doi:10.1186/s12964-020-00644-4)
Supplement: Supplementary file 3 — Additional file 2: Figure S1. HEK293T cells were cotransfected with GFP-LC3B and the plasmids containing the truncated P genes for 24 h, and further treated with CQ for 4 h. These cells were fixed, and immunostained with mouse anti-Flag antibodies (red), and then visualized using confocal microscopy. DAPI (blue) was used to stain nuclear DNA. Scale bar: 10 μm. The graph shows the quantification of autophagosomes by taking the average number of dots in 50 cells. Means and SD (error bars) of three independent experiments are indicated (*, P < 0.05. Figure S2. The truncated protein P5 is required for a ring-like structure. N2a cells were cotransfected with Flag and Myc tagged plasmids encoding the truncated P genes for 24 h, fixed, and immunostained with mouse anti-Flag antibody (green) and rabbit anti-Myc (red), and then visualized by confocal microscopy. DAPI (blue) stained nuclear DNA. Scale bar: 10 μm. Figure S3. Autophagosomes fail to fuse with lysosomes in Flag-P5-transfected cells. N2a cells were cotransfected with Flag-P5 and GFP-LC3B for 24 h, and were treated with EBSS or CQ for 4 h. Cells were fixed, and immunostained with rabbit anti-LAMP1 mAb (red), and mouse anti-Flag mAb (blue), and observed using confocal microscopy to analyze fusion of autophagosomes with lysosomes. Scale bar: 10 μm. The graph shows the quantification of autolysosomes by taking the average number of dots in 50 cells. Means and SD (error bars) of three independent experiments are indicated (*, P < 0.05; **, P < 0.01; ***, P < 0.001). Figure S4. The truncated P proteins colocalize with BECN1. N2a cells were cotransfected with the plasmids encoding the truncated P genes and Myc-BECN1 for 24 h, and Flag (green), BECN1 (red) and DAPI (blue) were detected by using the indicated antibodies in confocal microscopy. Scale bar: 10 μm. [file 12964_2020_644_MOESM3_ESM.pptx]

## Slide 1
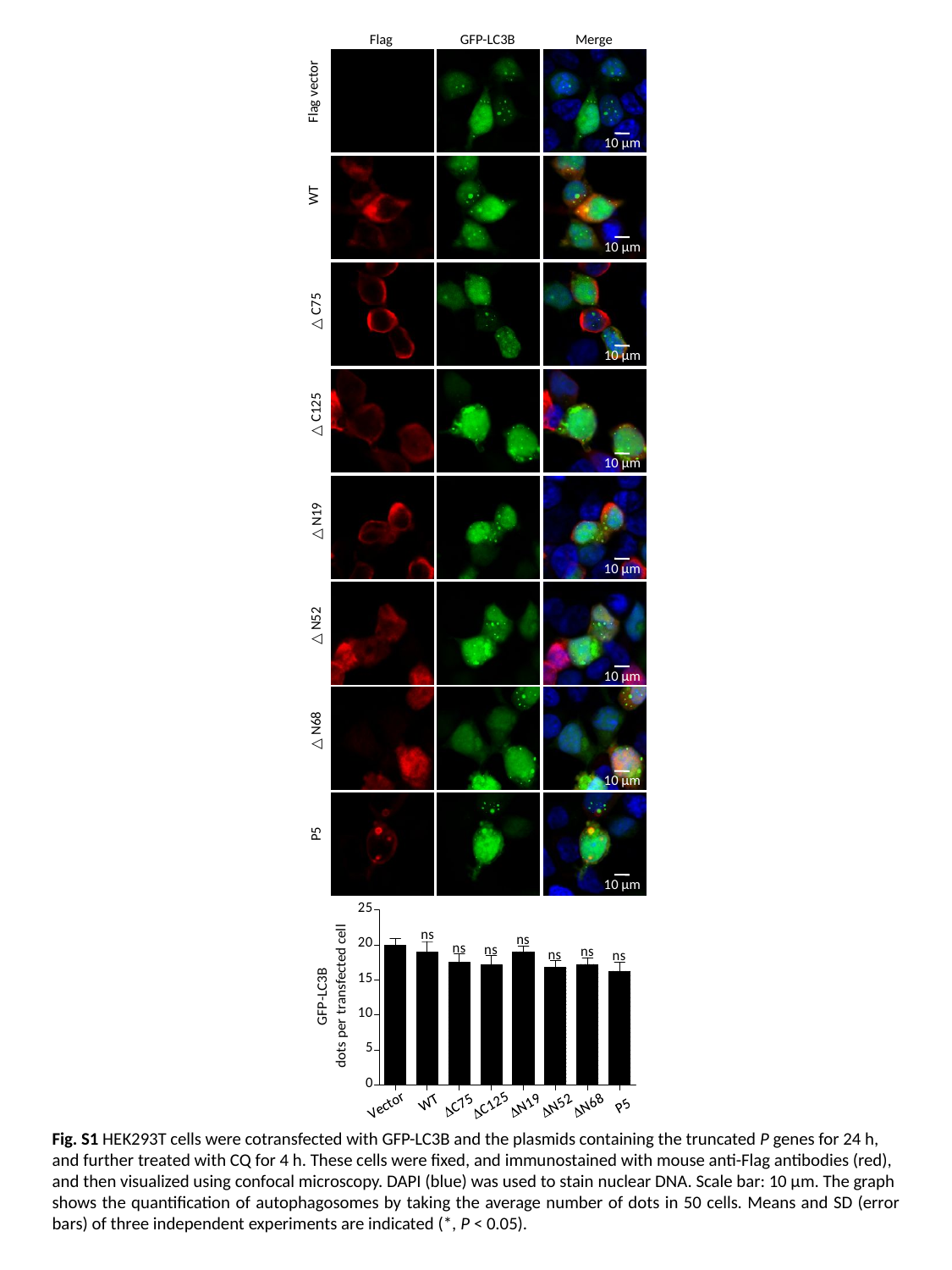

Flag
GFP-LC3B
Merge
Flag vector
10 μm
WT
10 μm
 △ C75
10 μm
 △ C125
10 μm
 △ N19
10 μm
 △ N52
10 μm
 △ N68
10 μm
 P5
10 μm
Fig. S1 HEK293T cells were cotransfected with GFP-LC3B and the plasmids containing the truncated P genes for 24 h,
and further treated with CQ for 4 h. These cells were fixed, and immunostained with mouse anti-Flag antibodies (red),
and then visualized using confocal microscopy. DAPI (blue) was used to stain nuclear DNA. Scale bar: 10 μm. The graph
shows the quantification of autophagosomes by taking the average number of dots in 50 cells. Means and SD (error bars) of three independent experiments are indicated (*, P < 0.05).

## Slide 2
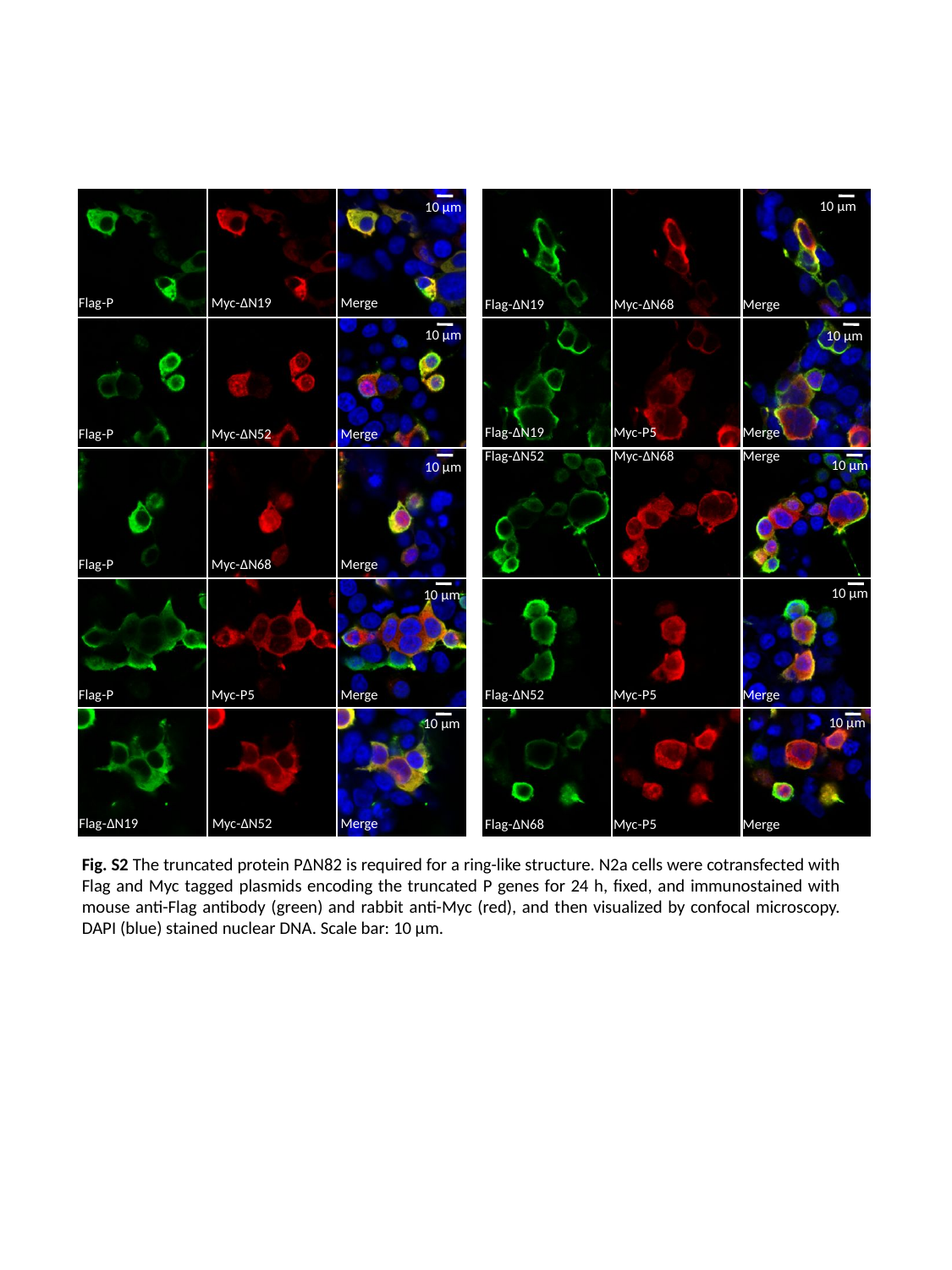

10 μm
10 μm
Flag-P
Myc-ΔN19
Merge
Flag-ΔN19
Myc-ΔN68
Merge
10 μm
10 μm
Flag-ΔN19
Myc-P5
Merge
Flag-P
Myc-ΔN52
Merge
Flag-ΔN52
Myc-ΔN68
Merge
Merge
10 μm
10 μm
Flag-P
Myc-ΔN68
Merge
10 μm
10 μm
Flag-ΔN52
Myc-P5
Merge
Flag-P
Myc-P5
Merge
10 μm
10 μm
Flag-ΔN19
Myc-ΔN52
Merge
Flag-ΔN68
Myc-P5
Merge
Fig. S2 The truncated protein PΔN82 is required for a ring-like structure. N2a cells were cotransfected with Flag and Myc tagged plasmids encoding the truncated P genes for 24 h, fixed, and immunostained with mouse anti-Flag antibody (green) and rabbit anti-Myc (red), and then visualized by confocal microscopy. DAPI (blue) stained nuclear DNA. Scale bar: 10 μm.

## Slide 3
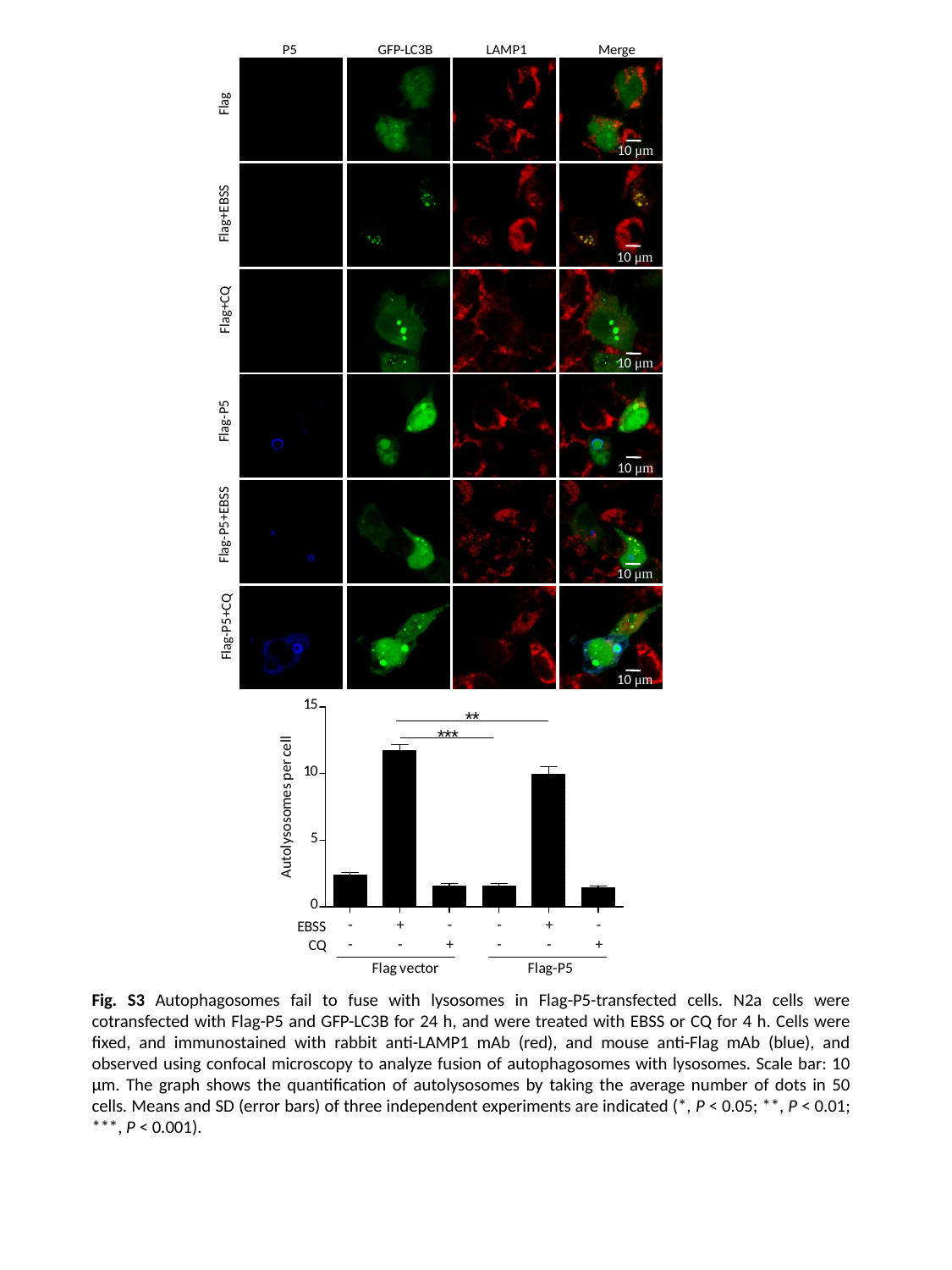

P5
GFP-LC3B
LAMP1
Merge
Flag
Flag+EBSS
Flag-P5
Flag-P5+EBSS
10 μm
10 μm
10 μm
10 μm
10 μm
10 μm
Flag+CQ
Flag-P5+CQ
Fig. S3 Autophagosomes fail to fuse with lysosomes in Flag-P5-transfected cells. N2a cells were cotransfected with Flag-P5 and GFP-LC3B for 24 h, and were treated with EBSS or CQ for 4 h. Cells were ﬁxed, and immunostained with rabbit anti-LAMP1 mAb (red), and mouse anti-Flag mAb (blue), and observed using confocal microscopy to analyze fusion of autophagosomes with lysosomes. Scale bar: 10 μm. The graph shows the quantification of autolysosomes by taking the average number of dots in 50 cells. Means and SD (error bars) of three independent experiments are indicated (*, P < 0.05; **, P < 0.01; ***, P < 0.001).

## Slide 4
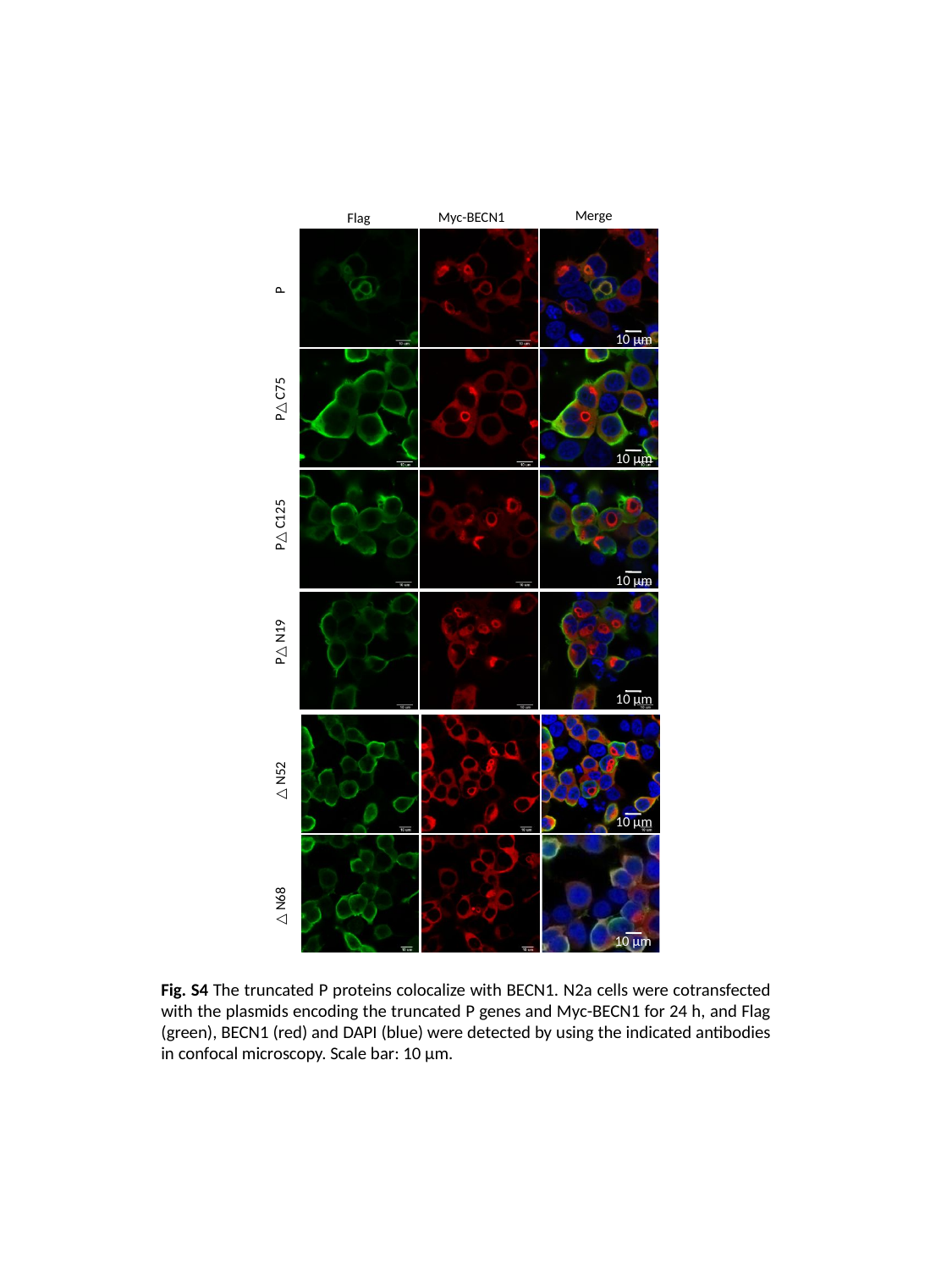

10 μm
 10 μm
 10 μm
Merge
Myc-BECN1
P
 10 μm
P△ C75
 10 μm
P△ C125
 10 μm
P△ N19
 10 μm
△ N52
 10 μm
△ N68
 10 μm
Flag
Fig. S4 The truncated P proteins colocalize with BECN1. N2a cells were cotransfected with the plasmids encoding the truncated P genes and Myc-BECN1 for 24 h, and Flag (green), BECN1 (red) and DAPI (blue) were detected by using the indicated antibodies in confocal microscopy. Scale bar: 10 μm.
